# A rare case of intracerebral hemorrhage complicating heparin-induced thrombocytopenia in a COVID-19 patient

**DOI:** 10.1016/j.amsu.2021.103070

**Published:** 2021-11-20

**Authors:** A. Zyani, K. Elyachioui, C. Treyi, M. Aabdi, H. Sbai

**Affiliations:** Anesthesiology and Intensive Care Unit, Faculty of Medicine and Pharmacy, Abdelmalek Essaadi University, Tangier, Morocco

**Keywords:** Heparin induced thrombocytopenia, Covid-19, Intracerebral hemorrhage

## Abstract

**Introduction:**

Heparin-induced thrombocytopenia is a rare complication of heparin therapy associated with thrombocytopenia and mainly thrombotic complications.

**Case report:**

we herein describe a case of a woman hospitalized for management of a severe case of COVID-19 treated with low molecular weight heparin, who developed heparin-induced thrombocytopenia complicated by intracerebral hemorrhage with no thrombotic complications.

**Conclusion:**

Discontinuation of heparin was effective without the use of other non-heparin anticoagulants, platelet transfusion or plasmapheresis.

## Highlights

• HIT is a rare complication of LMWH, it is associated with thrombocytopenia and mainly thrombotic complications.
• In this paper we will report a rare complication of HIT : ICH in a 63 years old woman admitted for covid 19-infection.
• Discontinuation of heparin was effective without the use of other non-heparin anticoagulants, platelet transfusion or plasmapheresis.

## 1 Introduction

In critically ill patients with covid-19, a systemic inflammatory response associated with endothelial activation is observed, causing different thrombotic complications, making the covid-19 infection a highly prothrombotic state [1].Therefore, thromboprophylaxis is strongly recommended in these patients, with some experts even advocating for therapeutic dosing to prevent thromboembolic events, a protocol used by our department as the screening of thrombo-embolic events remains limited.

Heparin-induced thrombocytopenia (HIT) is a severe, life-threatening drug reaction associated with a decrease in platelet count and a high risk of thrombosis caused by platelet-activating antibodies against platelet factor 4/heparin complexes [2].

HIT may be aggravated by life-threatening arterial and venous thrombosis and, to a lesser extent, hemorrhagic complications [3].

In this paper, we will report the case of a probable HIT associated with covid 19 infection complicated with intracerebral hemorrhage (ICH).

## 2 Case report

A 63-year-old women, diabetic with no other medical history was admitted to the intensive care unit for COVID 19 infection. A thoracic computed tomography scan found lesions classified as CORADS 5 with an estimated lung damage of 70% and positive nasopharyngeal reverse-transcriptase–polymerase chain reaction for Covid-19.

Upon her admission, the patient was put on vitamin therapy, antibiotics, corticosteroids and high dose anticoagulation with low molecular weight heparin 100 UI/kg/12h.

**Fig. 1:**
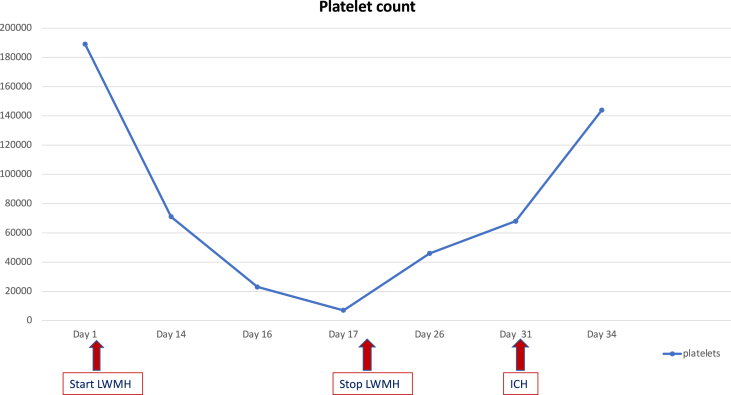
Trends of platelets over the patient's hospitalization.

The initial complete blood count was normal with platelet count of 188 000/mm3, a hemoglobin of 12,3 g/dl and WBCs at 5960/mm3. A control assessment carried out 14 days later showed a thrombocytopenia at 71 000/mm3; a hemoglobin of 12.6 and WBCs at 23 000/mm3 (Fig. 1).

The diagnosis of heparin-induced thrombocytopenia was suspected: her 4Ts score was at 6 indicating a high probability of HIT: 2 points for thrombocytopenia (>50% fall), 2 for timing of platelet count fall (Onset with 5–10 days of exposure to heparin), 0 for thrombosis (none) and 2 for no other cause for thrombocytopenia identified.

Confirmation of the diagnosis by HIT-antibody testing or platelet activation testing was not possible due to the unavailability of these tests in our region.

The LMBW was discontinued and the patient kept lowering her platet count numbers to a minimum of 13 000/mm3.

14 days later, the patient presented with neurological deficit made of a right hemiparesis with a muscular strength rated at 4/5 associated with a platelet count of 68 000/mm3.

**Fig. 2:**
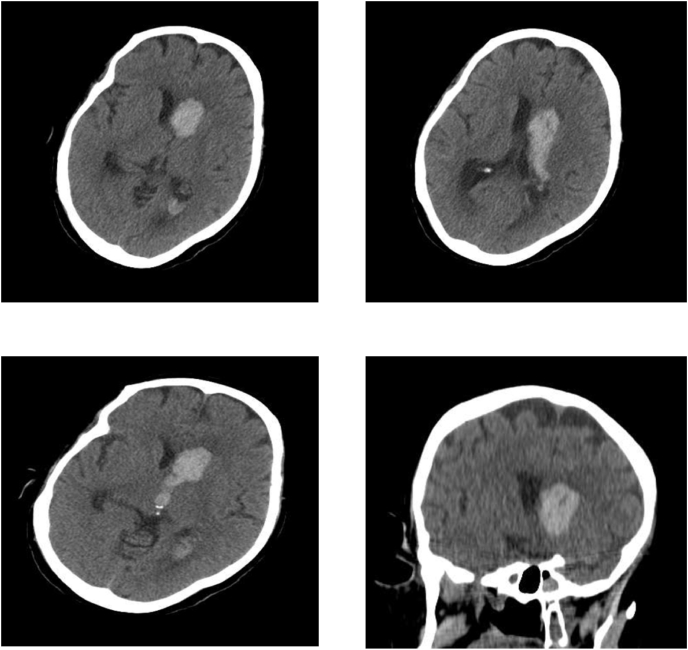
Axial non contrast brain computed tomography demonstrating a large parietal hyperdense hemorrhage measuring 40 × 15mm surrounded by a small area of peri-lesional edema with compression of the homolateral lateral ventricle associated with an intra ventricular hemorrhage.

A brain CT scan was performed showing an intracerebral hemorrhage associated with intra ventricular hemorrhage (Fig. 2).

Afterwards, the patient improved on respiratory, neurological and biological levels, without platelets transfusion or plasmapheresis, allowing her to be discharged from the ICU.

## 3 Discussion

In HIT, the underlying physiology is the formation of a heparin-dependent anti-platelet factor immune complex resulting in immunologic platelet activation and thrombin generation, and subsequent thrombocytopenia and thrombotic risk [[4], [5], [6]].

In a series of 6332 patients hospitalized with heparin-induced thrombocytopenia, bleeding was seen in 5.7% of patient (gastrointestinal bleeding being the most common) compared to thrombotic events which was found in 20.7% of patients [7].

Hemorrhagic complications due to HIT are rare.Most of them are related with adrenal hemorrhage which is linked to adrenal vein thrombosis that is further complicated by arterial hemorrhage [[8], [9], [10], [11]]and intra tumor hemorrhage [12].

Neurologic complications associated with HIT are relatively rare *. Pohl.C and al* found in a retrospective study of 120 patients with HIT that only 9.2% of them had neurologic complications and included mainly cerebrovascular ischemia and cerebral veinous thrombosis.Primary intracerebral hemorrhage was not observed [13].

*Hertle DN and al* were first to report a case of HIT possibly leading to ICH in a neurosurgical patient [14].

*Tun NM and al* reported the first confirmed case of HIT that was first complicated by ICH and later by deep vein thrombosis [15].

Other reported cases of ICH complicating HIT were then reported [16].

American Society of Hematology guidelines recommends the use of 4T score to estimate the pretest probability of HIT [3]. In those with intermediate or high pretest probability, testing for the presence of HIT antibodies should be done, by using immunoassays or platelet activation assays [17]. In our case, laboratory testing in order to confirm the diagnosis by heparin-PF4 antibody testing or platelet activation tests was impossible due to the absence of these tests in our region and our inability to perform the test elsewhere.It should be noted that antibody testing in covid-19 patients may be falsely positive due to a strong reactivity in PF4/heparin antigen tests found in these patients without the presence of platelet-activating antibodies.Functional testing are then required to confirm the diagnosis [18].

Regarding HIT in COVID patients,a retrospective study reviewed 7 cases of HIT in critically-ill COVID-19 patients.All patients presented antibodies to PF4/heparin. Diagnosis was confirmed for the 7 patients using the heparin-induced platelet aggregation test. All the patients received curative dose of LMWH then unfractionated heparin. Five patients experienced at least 1 severe clinical thromboembolic event, no hemorrhagic complication was observed [19].

COVID-19 is now known to be associated with thrombocytopenia,with an incidence that varies between 5 and 41.7% [20]. A meta-analysis of over 7163 patients showed that thrombocytopenia might be a risk factor for developing a severe state [21].Interestingly, there is no data on the role of thrombocytopenia in increasing the risk of bleeding in COVID-19.

Regarding the management of highly suspected or confirmed HIT, the first step is to stop heparin and to initiate a non-heparin anticoagulant for the prevention of thrombotic events [2]. Available options for anticoagulation include argatroban, bivalirudin, danaparoid, fondaparinux, or a direct oral anticoagulant [3].No alternate anticoagulant was used in order to prevent the worsening of the ICH.

## 4 Conclusion

Critically ill patients with COVID-19 develop life threatening coagulopathy and thromboembolic complications that justify aggressive anticoagulation. However, the occurrence of HIT and the possibility of COVID-19 being an independent risk factor for HIT,increases the risk of severe thrombotic events and could alter the risk–benefit balance of anticoagulation [22].

## Ethical approval

This study was exempt from ethical approval at our institution, as it was an observational finding in regular practice.

## Sources of funding

None.

## Authors’ contributions

Dr. ZYANI Adil contributed to conceptualization, methodology, validation, formal analysis, and visualization and wrote the original draft.

Dr. EL YACHIOUI Khalil contributed and reviewed and edited the manuscript.

Dr. TREYI Chaymae provided resources and developed software and was responsible for data curation.

Dr AABDI Mohammed reviewed and edited the manuscript.

Pr. SBAI Hicham was responsible for project administration; provided resources; contributed to conceptualization, methodology, and validation; and reviewed and edited the manuscript.

## Registration of research studies

As this manuscript was a case report with no new medical device nor surgical techniques, not prior registration is required.

## Consent

Written informed consent was obtained from the patient for publication of this case report.

## Guarantor

ZYANI Adil.

EL YACHIOUI Khalil.

## Provenance and peer review

Not commissioned, externally peer reviewed.

## Data availability

The data used to support the findings of this study are available from the corresponding author.

## Declaration of competing interest

The authors declare no conflicts of interest.

A rare case of intracerebral hemorrhage due to heparin-induced thrombocytopenia in a COVID-19 patient.
